# A novel variant in the *SLCO2A1* gene in a Chinese patient with chronic gastroenteropathy and primary hypertrophic osteoarthropathy

**DOI:** 10.1186/s13023-024-03221-x

**Published:** 2024-06-11

**Authors:** Yimin Dai, Miao He, Hui Xu, Bei Tan, Weixun Zhou, Wei Liu, Qiang Wang, Jingyi Huang, Qing Shang, Yaping Liu, Yue Li

**Affiliations:** 1 Department of Gastroenterology, Peking Union Medical College Hospital, Chinese Academy of Medical Sciences and Peking Union Medical College, No. 1, Shuaifuyuan, Beijing, 100730 China; 2 McKusick-Zhang Center for Genetic Medicine, State Key Laboratory of Medical Molecular Biology, Institute of Basic Medical Sciences, Chinese Academy of Medical Sciences and Peking Union Medical College, Beijing, China; 3 Department of Pathology, Peking Union Medical College Hospital, Peking Union Medical College Hospital, Chinese Academy of Medical Sciences & Peking Union Medical College, Beijing, 100730 China; 4 Department of Radiology, Peking Union Medical College Hospital, Peking Union Medical College Hospital, Chinese Academy of Medical Sciences & Peking Union Medical College, No. 1, Shuaifuyuan, Beijing, 100730 China; 5 The State Key Laboratory for Complex, Severe, and Rare Diseases, The State Key Sci-tech Infrastructure for Translational Medicine, Peking Union Medical College Hospital, Beijing, 100730 China

**Keywords:** Chronic enteropathy associated with *SLCO2A1* gene, Primary hypertrophic osteoarthropathy, DNA sequencing, Nonsense-mediated mRNA decay

## Abstract

**Background:**

Chronic enteropathy associated with *SLCO2A1* gene (CEAS) results from loss-of-function variants in *SLCO2A1*, which encodes the prostaglandin transporter (PGT). CEAS follows an autosomal recessive inheritance pattern. To date, approximate 30 pathogenic variants have been reported in CEAS.

**Methods:**

We performed whole exome sequencing (WES) to screen for potential pathogenic variants in a patient suspected of having CEAS, and confirmed a variant in *SLCO2A1* using Sanger sequencing. We established an in vitro minigene model to compare splicing between wild type (WT) and mutant transcripts. Quantitative polymerase chain reaction (qPCR) was used to evaluate *SLCO2A1* transcription in the stomach and colon tissues from the patient and a healthy control (HC). The transcripts were further cloned and sequenced.

**Results:**

The patient had a novel, homozygous, recessive c.929A > G variant in exon 7 of *SLCO2A1*, which has not been previously reported in CEAS or PHO. This variant altered splicing, resulting in an exon 7‐truncated transcript lacking 16 bases. No normal transcript was detected in the patient’s stomach or colon tissue. qPCR also showed significantly decreased *SLCO2A1* transcription compared to HC.

**Conclusion:**

A previously unreported variant caused defective *SLCO2A1* splicing and reduced mRNA levels in a patient with CEAS and PHO. This research enhances understanding of CEAS and PHO pathophysiology and aids genetic counseling and diagnosis.

**Supplementary Information:**

The online version contains supplementary material available at 10.1186/s13023-024-03221-x.

## Introduction

Chronic enteropathy associated with the *SLCO2A1* gene (CEAS) was initially described by Umeno et al., as a disease characterized by chronic nonspecific multiple ulcers in the small intestine, associated with pathogenic variants in the *SLCO2A1* gene and inherited in an autosomal recessive pattern [1]. CEAS patients mainly manifest intestinal ulcers and thickening of intestinal folds, along with persistent hypoalbuminemia and blood loss. In some cases, the upper digestive tract may also be affected [1]. In addition to CEAS, the *SLCO2A1* gene is also the pathogenic gene for primary hypertrophic osteoarthropathy (PHO) type 2, characterized by periostosis, pachydermia and digital clubbing [2]. It is possible for patients with *SLCO2A1* variants to concurrently experience both CEAS and PHO. According to Hong et al., 14 out of 46 patients (30.4%) with chronic intestinal ulcers were genetically diagnosed with CEAS, and among those, 5 CEAS patients also exhibited PHO [3]. Additionally, there is a significant gender disparity between CEAS and PHO. CEAS affects a higher proportion of females, with a male-to-female ratio of 1:2.5 to 1:3 [4], whereas PHO shows a male predominance, with a ratio of nearly 7:1 [5]. Males also tend to be more prevalent among patients who have both CEAS and PHO [6].

The *SLCO2A1* gene encodes a transmembrane prostaglandin transporter (PGT) that plays a crucial role in prostaglandin E2 (PGE2) metabolism. Variants in this gene lead to transporter dysfunction and subsequent accumulation of PGE2 [2]. To date, nearly 100 pathogenic variants have been identified in PHO and CEAS patients, with varying pathogenic effects [4]. Among these, the most frequently reported is c.940 + 1G > A, which affects gene splicing and leads to a frameshift [1]. Additionally, several variants within exon regions are missense and nonsense predicted to be pathogenic by software [1, 7, 8]. However, experimental evidence supporting the precise pathogenicity of most *SLCO2A1* variants has been limited.

In this study, we report a novel homozygous *SLCO2A1* variant in a 32-year-old male patient with CEAS and PHO. We performed Sanger sequencing, mRNA analysis and minigene assays to comprehensively uncover the pathogenic mechanism of this variant.

## Methods

### Patient and samples

This research involved a patient who was suspected to have both PHO and CEAS as diagnosed by specialist physicians from the department of endocrinology and the department of gastroenterology. We selected a healthy adult male as a control (HC). Written informed consents were obtained from the patient and the HC. This study received ethics approval from the hospital’s Ethics Committee (S_K1478) and was conducted in accordance with the Declaration of Helsinki. After obtaining written informed consents, we collected blood samples from the patient for whole-exome sequencing and Sanger sequencing. We also collected a blood sample from the patient’s mother for hereditary verification. Additionally, we obtained gastric and colonic tissues via endoscopy for immunohistochemical staining to assess PGT expression.

### Whole-exome sequencing

Genomic DNA was extracted from the patient’s peripheral blood using the QIAamp DNA Blood Midi kit (Qiagen). For whole-exome sequencing (WES) analysis, KingMed Diagnostics (Beijing, China) employed an Illumina Genome Analyzer to identify variants. To validate the WES results, Sanger sequencing was carried out using the following primers:

- Forward Primer: 5’-CTGAGGCCAGAGACTCAAGG-3’
- Reverse Primer: 5’-AGAGATGCATGGAAACCCTGTG-3’

The reference sequence (NM_005630) was obtained from the University of California Santa Cruz human genome assembly 19 (UCSC.hg19; genome.ucsc.edu/) and National Center for Biotechnology Information (https://www.ncbi.nlm.nih.gov/gene/6578).

### Minigene assay

**Fig. 1:**
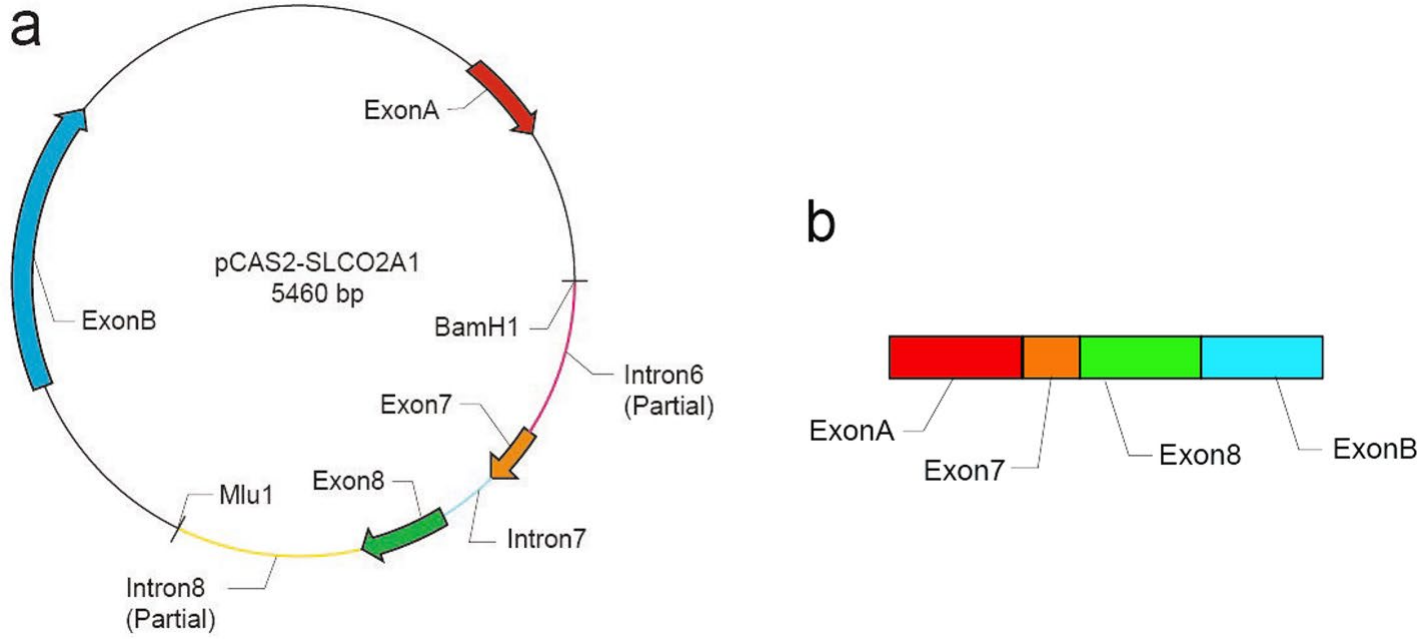
Structure of the *SLCO2A1* minigene construct. **a** Exon 7 (orange arrow) carrying variant site, the neighboring exon 8 (green arrow), intron 7 between the two exons as well as part of intron 6 and 8 were amplified and inserted into the plasmid pCAS2, forming the minigene which could be transcribed. **b** Schematic diagram of the mRNA of the minigene. The mRNA contained exon 7 and 8 from *SLCO2A1* and exon A and B from pCAS2

To assess whether the variant affects *SLCO2A1* gene splicing, we employed a minigene assay [9]. A minigene model was constructed, encompassing sequences from the middle of intron 6 to the middle of intron 8, including the 5′ and 3′ regions of the *SLCO2A1* gene. This design incorporated the identified c.929A > G variant in exon 7. Primers with homologous arms for the BamH1 and MluI restriction endonucleases (New England Biolabs) were utilized to merge the minigene segment with an expression vector through In-Fusion Cloning (TaKaRa; https://www.takarabio.com/learning-centers/cloning/in-fusion-cloning-tools). During construction, the minigene was transcribed into cDNA, which contained exon 7 and 8, as well as exon A and B from the pCAS2 reporter vector, followed by ligation (Fig. 1).

The target *SLCO2A1* sequence was amplified by polymerase chain reaction (PCR) with the previously described primers. The PCR product was purified via gel extraction (QIAGEN Biolabs) and ligated into the pCAS2 reporter vector (provided by Mario Tosi, Rouen Institute for Biomedical Research). This vector was pre-digested with BamH1 and MluI to facilitate construction of the recombinant plasmid. Following transformation into *Escherichia coli*, plasmids were extracted from monoclonal colonies and sequenced to confirm the successful construction and transformation of the vector. The verified plasmids were then transfected into HEK293 cells (Cell Resource Center, Peking Union Medical College) using Lipofectamine 3000 (Thermo Fisher Scientific). At 24 h post-transfection, RNA was extracted for analysis by quantitative real-time polymerase chain reaction (qPCR). The qPCR products were subsequently sequenced and analyzed via gel electrophoresis to visualize and identify any transcriptional alterations resulting from the variant plasmid.

### *SLCO2A1* transcription levels in tissues

To evaluate *SLCO2A1* expression in tissues, gastric antrum and sigmoid colon mucosal biopsies were collected from both the patient and a healthy control (HC) during endoscopy procedures. Total RNA was extracted from these biopsies, and mRNA levels of *SLCO2A1* were assessed using qPCR with the following primers:

- Forward Primer: 5’-ATGAGGATCGAGACTATC-3’
- Reverse Primer: 5’-TAACGTGCATGGTCGCCTGTG-3’

To ensure the reliability of the results, qPCR was conducted in triplicate to minimize the potential for interference.

### T-A cloning for transcript analysis

We performed T-A cloning to investigate potential differences in the predominant *SLCO2A1* transcripts between the patient and HC tissues. Total RNA was extracted from the tissues and then reverse transcribed into cDNA corresponding to the *SLCO2A1* target sequences. Subsequently, the cDNA was ligated into T vectors and transformed into competent *Escherichia coli*. From each of the patient and HC samples, we selected fifty individual colonies separately from the patient and the HC. Sanger Sequencing was then carried out on these colonies to identify any significant transcript variations. If no mRNA was detected, no sequencing signal would be generated, indicating the absence of a transcript.

### Immunohistochemical staining

We conducted Immunohistochemical staining (IHC) on gastrointestinal mucosal tissues obtained from both the patient and the HC to examine the expression and localization of the target protein. For this purpose, we utilized the following primary antibodies: SLCO2A1 (rabbit, HPA013742, Sigma-Aldrich, St. Louis, MO), and CD31 (mouse, PA0414, LEICA, Chicago, IL). CD31 was chosen to label the mucosal vessel cells in the stomach and colon. The stained tissue sections were independently assessed by two pathologists.

## Results

### Clinical manifestations

The patient, a 32-year-old male with non-consanguineous parents, presented with a complex medical history. He had been experiencing skin hardening and thickening for the past 10 years. Additionally, over the past 4 years, he had been intermittently suffering from episodes of bloody and purulent stools. Notably, the patient’s mother was in good health, but his father had passed away in his 50 s due to cholangiocarcinoma. Importantly, there was no pathogenic manifestations of PHO, CEAS or inflammatory bowel disease (IBD) in any other members of the patient’s family.

**Fig. 2:**
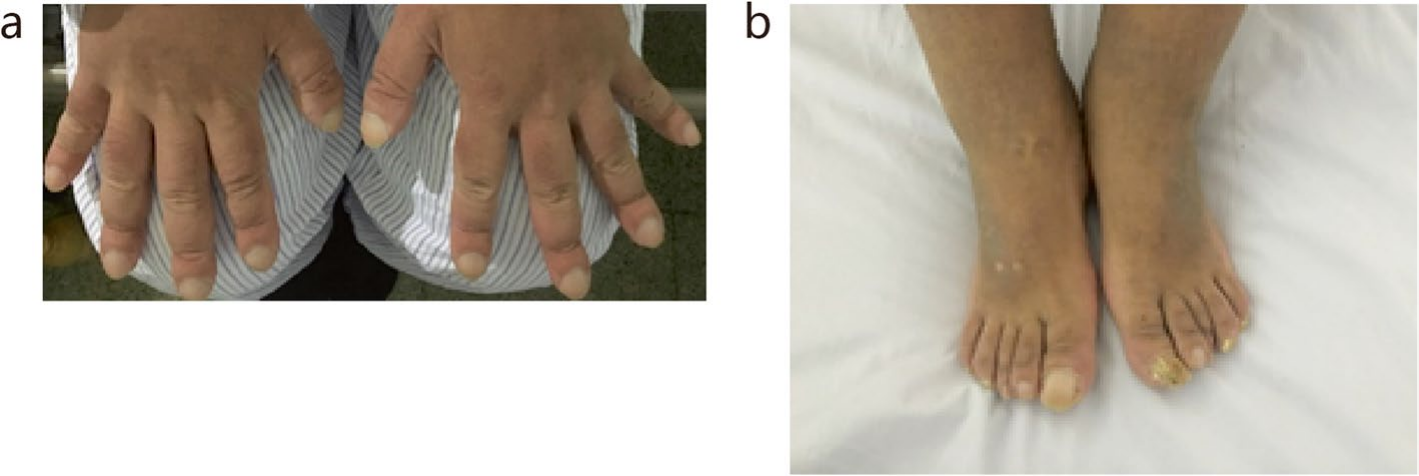
Clinical manifestations of the patient. **a** & b Finger clubbing of the hands and feet, indicating digital clubbing

**Fig. 3:**
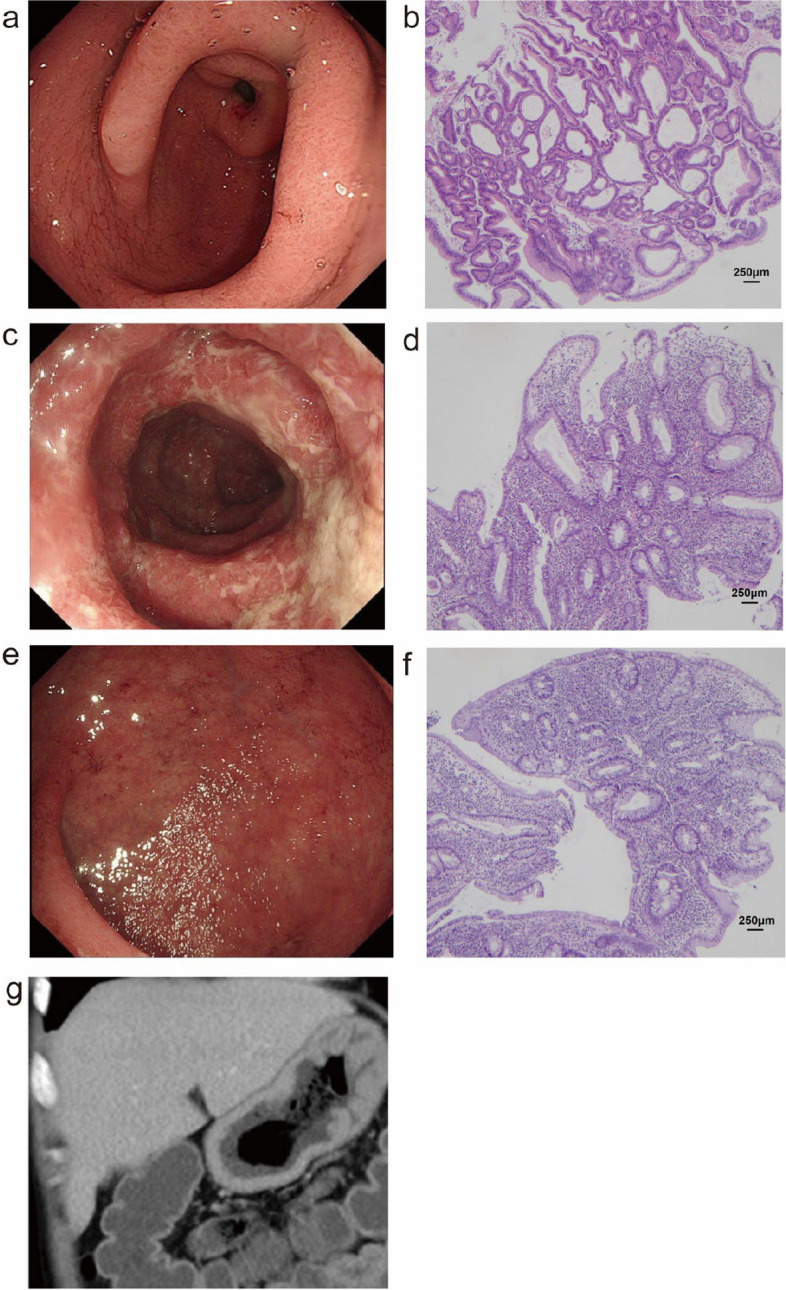
Gastrointestinal endoscopy, imaging, and pathological findings of the patient. **a** Esophagogastroduodenoscopy showing thickened, coarse stomach folds and nodular eminences. **b** Hematoxylin and eosin (H&E) staining of antrum biopsyshowing enlarged, densely packed glands (200× magnification). **c **& **e** Colonoscopy showing multiple irregular superficial ulcers, diffuse congestive edema and purulent exudation of the sigmoid colon and the rectum. **d** H&E staining of the sigmoid colon showing inflammation and disrupted crypt architecture (200× magnification). **f** H&E staining of the rectum also showing disrupted crypts. **f** Contrast-enhanced abdominal computed tomography scan demonstrating thickened stomach folds and increased mucosal enhancement in the sigmoid and rectum

At the age of 22, the patient began to experience gradual thickening and hardening of the scalp and facial skin, accompanied by the development of wrinkles. Additionally, he exhibited finger and toe thickening with digital clubbing (Fig. 2a & b). X-rays of the limbs showed periostosis, cortical thickening and diffuse increased bone density. Four years later, at the age of 26, the patient’s condition had evolved. He developed recurrent lower abdominal pain, as well as bloody and purulent stools occurring 2–3 times daily, which later increased to 4–5 times daily. Clinical examination showed mild hypoalbuminemia (31–34 g/L) and anemia (93–111 g/L). Notably, the Erythrocyte sedimentation rate (ESR) was elevated at 55 mm/h (normal range: 0-15 mm/h), and the high-sensitivity C-reactive protein (hsCRP) was elevated at 23.41 mg/L (normal: < 8.00 mg/L). Esophagogastroduodenoscopy revealed significant thickening of gastric folds in the cardia, fundus, body, antrum, and the descending duodenum (Fig. 3a & Supplementary Fig. 1). Hematoxylin and eosin (H&E) stained gastric biopsies showed enlarged, densely packed glands (Fig. 3b). The colonoscopy findings included diffuse mucosal edema, erosions, and purulent exudate in the sigmoid colon and rectum (Fig. 3c & e). Colonic and rectal H&E stains showed inflammation and disrupted crypt architecture, consistent with ulcerative colitis (UC) (Fig. 3d & f). Computerized tomography enterography (CTE) revealed gastric and duodenal wall thickening and enhancement, a normal small intestine, and an enhanced, thickened wall of the sigmoid colon and rectum (Fig. 3g). Clinically, the patient received diagnoses of hypertrophic gastritis, UC, and was suspected to have PHO. During his hospital stay, the patient’s frequent mucopurulent stools gradually transitioned to yellow pasty stools, and the frequency of bowel movements decreased to 1–2 times a day after treatment with oral mesalazine and hydrocortisone enema. He maintained remission with the use of mesalazine until experiencing a relapse nearly 3 years later. During the relapse, the colitis extended to involve the entire colon and rectum (Supplementary Fig. 2).

### Identification of a homozygous variant in *SLCO2A1* gene

**Fig. 4:**
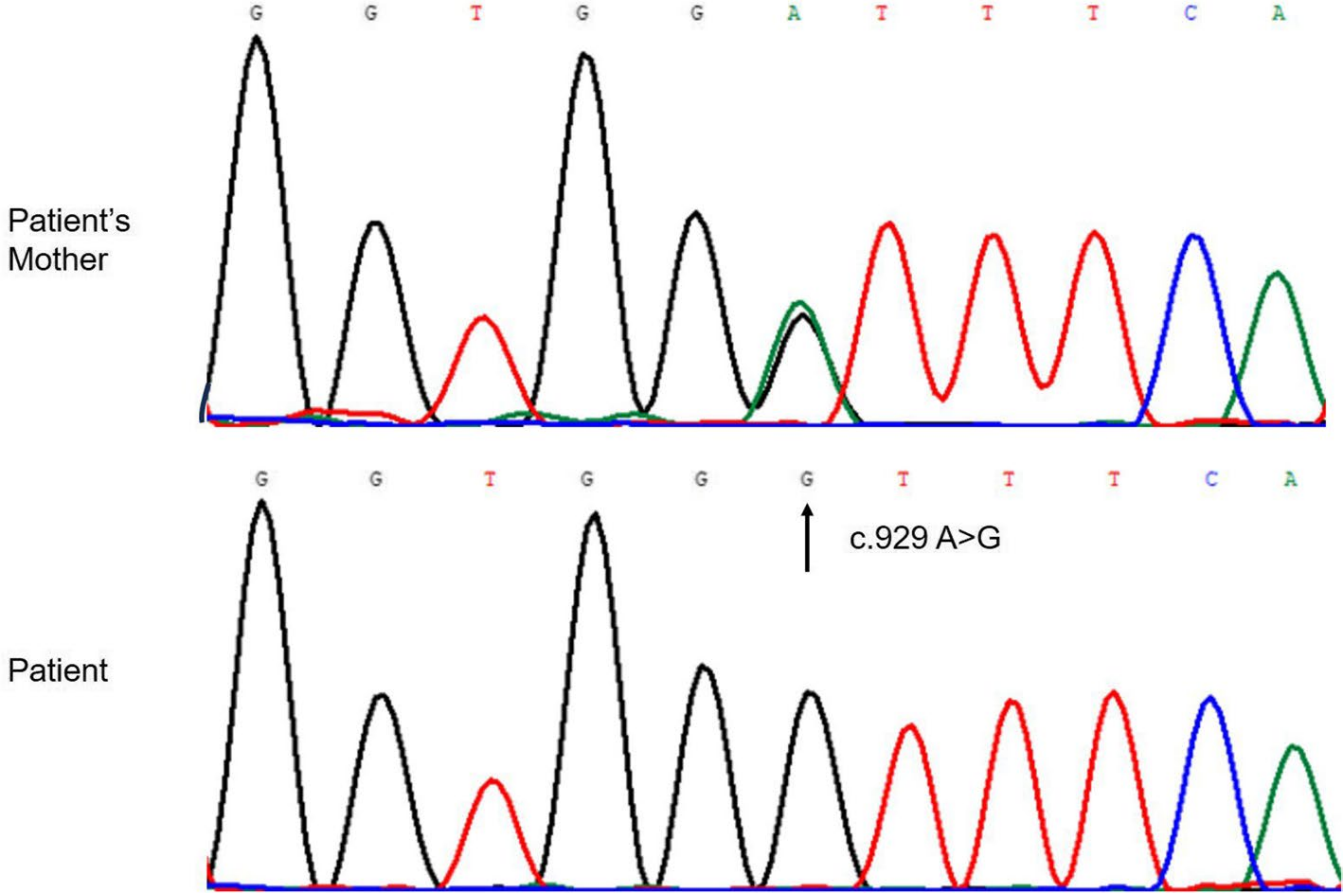
Sanger sequencing chromatograms of exon 7 of the *SLCO2A1 *gene. The patient (bottom) showed a homozygous A>G variant (c.929A>G) represented by the black peak. The patient’s mother (top) exhibited a heterozygous variant, with overlapping black and green peaks indicating both wildtype and variant alleles

WES identified the presence of a previously unreported homozygous c.929A > G variant in the *SLCO2A1* gene in the patient. Subsequently, Sanger sequencing was conducted to confirm the WES findings. Genetic analysis showed that the patient’s mother was a heterozygous carrier of this variant (Fig. 4), indicating that the variant was inherited maternally. Unfortunately, the father’s genotype could not be determined due to his early death.

The c.929A > G variant is situated at the exon 7 boundary of the *SLCO2A1* gene. In silico predictions from Mutation Taster and Polyphen-2 indicated that this novel variant was potentially pathogenic. These predictions indicated a likelihood of amino acids changes and alterations in protein features. It’s important to note that the predictions did not rule out the possibility of effects on splicing as well.

### Splicing variant revealed by minigene assay

**Fig. 5:**
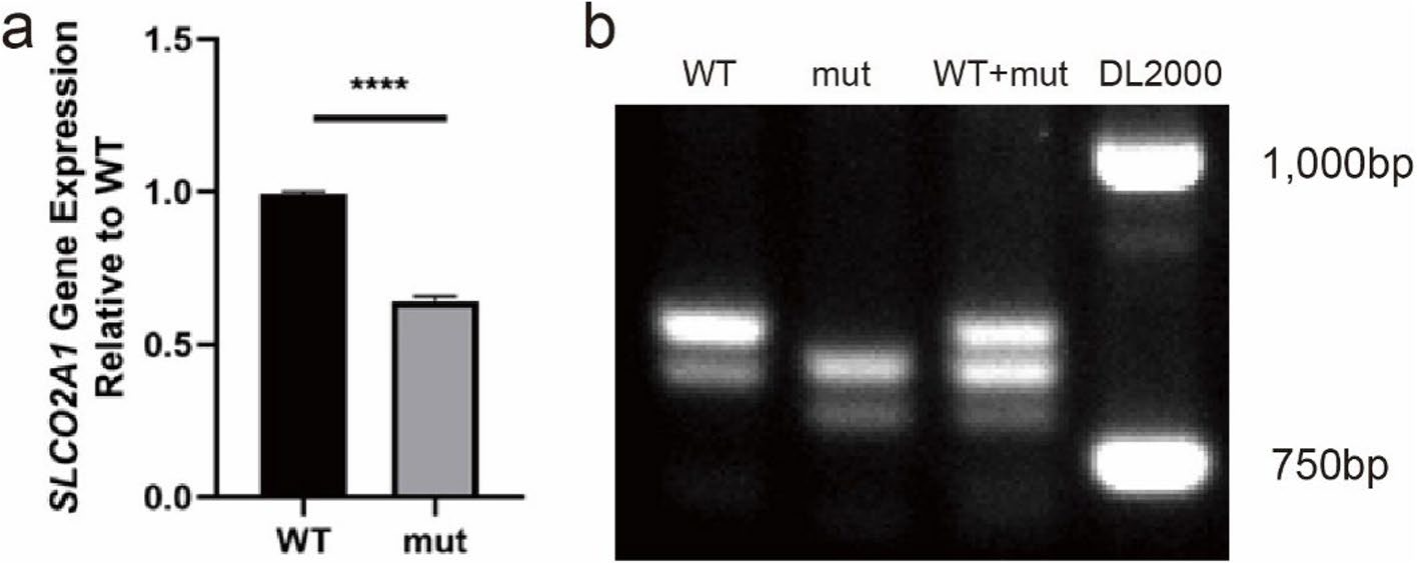
Minigene splicing assay. **a** qPCR analysis of minigene transcripts in HEK-293 cells transfected with patient or healthy control (HC) *SLCO2A1* minigene constructs. The patient’s minigene containing the c.929A>G variant (mut) showed significantly lower expression compared to the HC minigene (WT). **b** Gel electrophoresis of the RT-PCR products showing the patient minigene transcript (mut) was significantly shorter than the HC minigene transcript (WT). Faint shorter bands in both the patient and HC lanes indicate low levels of alternatively spliced transcripts

**Fig. 6:**
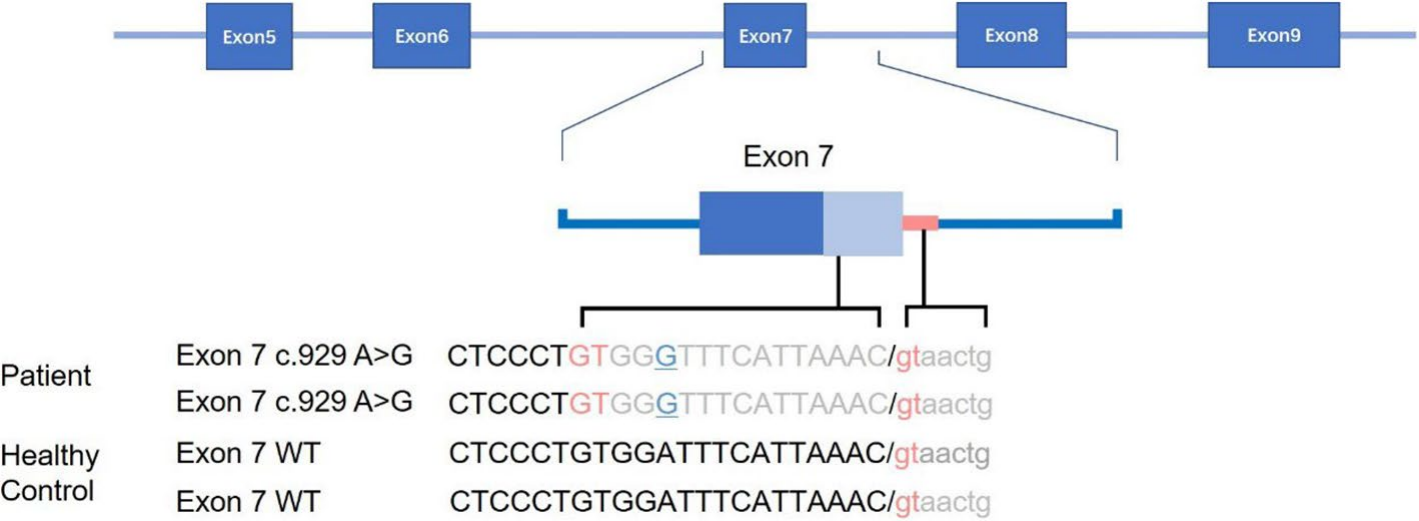
Schematic diagram of the splicing defect caused by the c.929A>G variant. Sanger sequencing revealed the patient’s exon 7 lacks 16 bases pairs compared to the HC (the blue underlined ‘G’ indicates the c.929A>G variant location). In the HC, the splice donor site (red lowercase ‘gt’) is located 16 base pairs downstream from the variant site. In the patient, the c.929A>G variant causes the splice donor site to shift upstream (red capital ‘GT’), leading to the exclusion of 16 base pairs from exon 7 during splicing

Subsequent qPCR analysis of the constructed minigene demonstrated a noteworthy reduction in transcription levels in the patient compared to the healthy control (Fig. 5a). Gel electrophoresis of the RT-PCR products further illustrated that the patient’s transcript was significantly shorter than that of the HC (Fig. 5b). Additionally, upon Sanger sequencing of the RT-PCR products, a 16-base pair deletion was identified in the transcripts originating from the variant-containing minigene when compared to those from the HC (Fig. 6).

### Decreased *SLCO2A1* expression

**Fig. 7:**
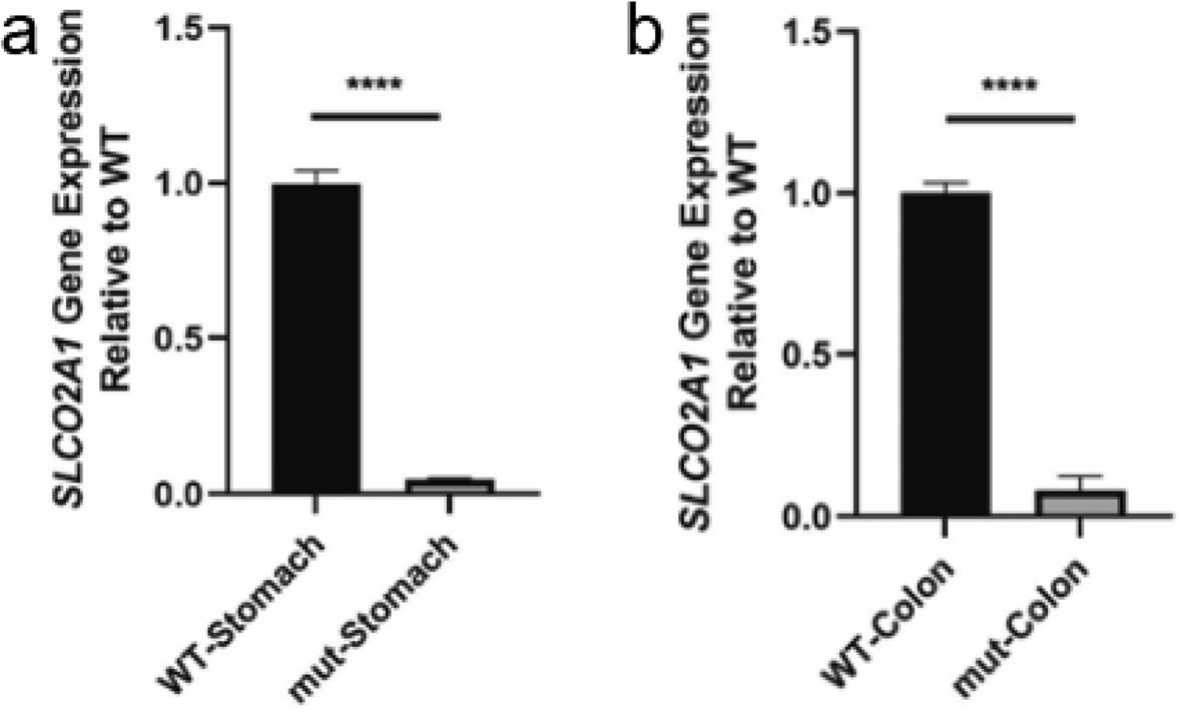
qPCR analysis of *SLCO2A1* expression in patient and healthy control (HC). **a** *SLCO2A1* mRNA levels were reduced in stomach tissure from the CEAS patient compared to HC stomach tissue. **b ***SLCO2A1* mRNA levels were also decreased in colon tissue from the CEAS patient relative to HC colon tissue

**Fig. 8:**
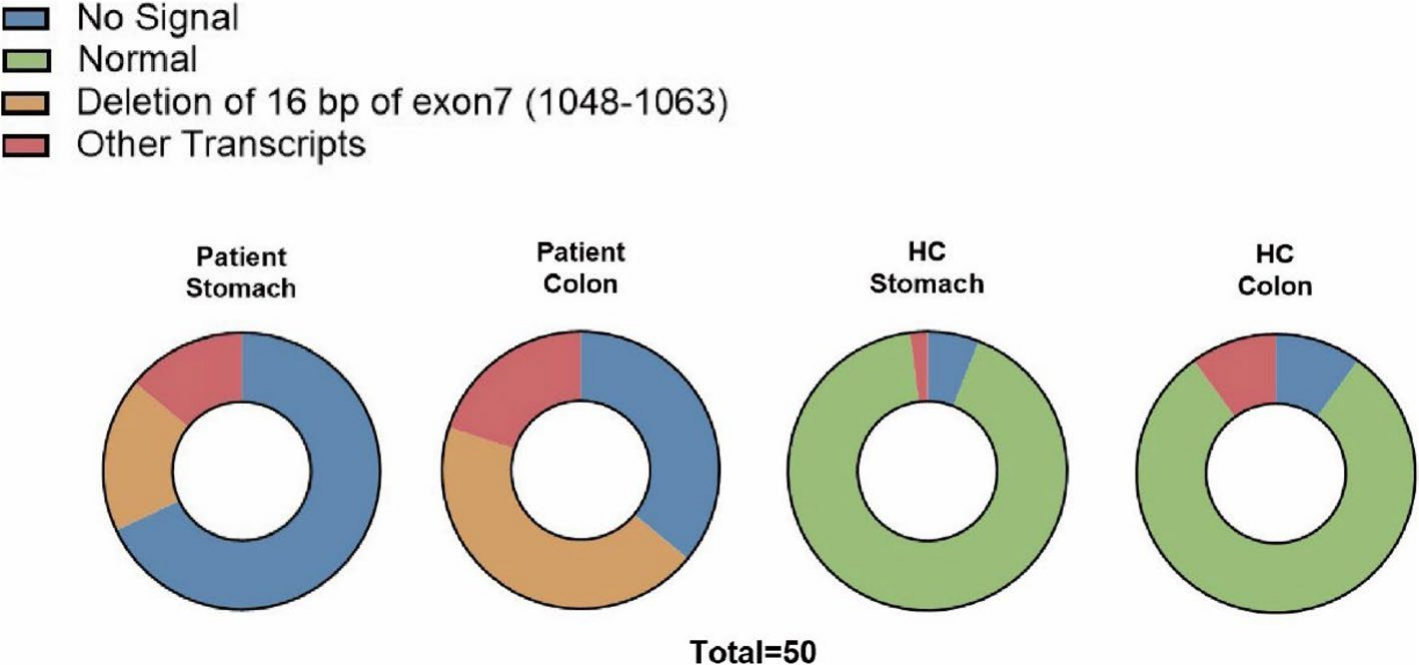
TA cloning analysis of *SLCO2A1 *transcripts in patient and healthy control (HC) tissues. RNA was extracted from stomach and colon tissues from the CEAS patient and HC. Sanger sequencing of cloned TA products revealed: the patient’s stomach and colon tissues lacked normal *SLCO2A1 *transcripts. The major transcript species had a 16 bp deletion in exon 7, consistent with the minigene assay results. HC stomach and colon tissues primarily contained normal *SLCO2A1 *transcripts. Many of the patient’s clones showed no transcript signal, indicating the c.929A>G variant likely causes nonsense-mediated mRNA decay (NMD)

**Fig. 9:**
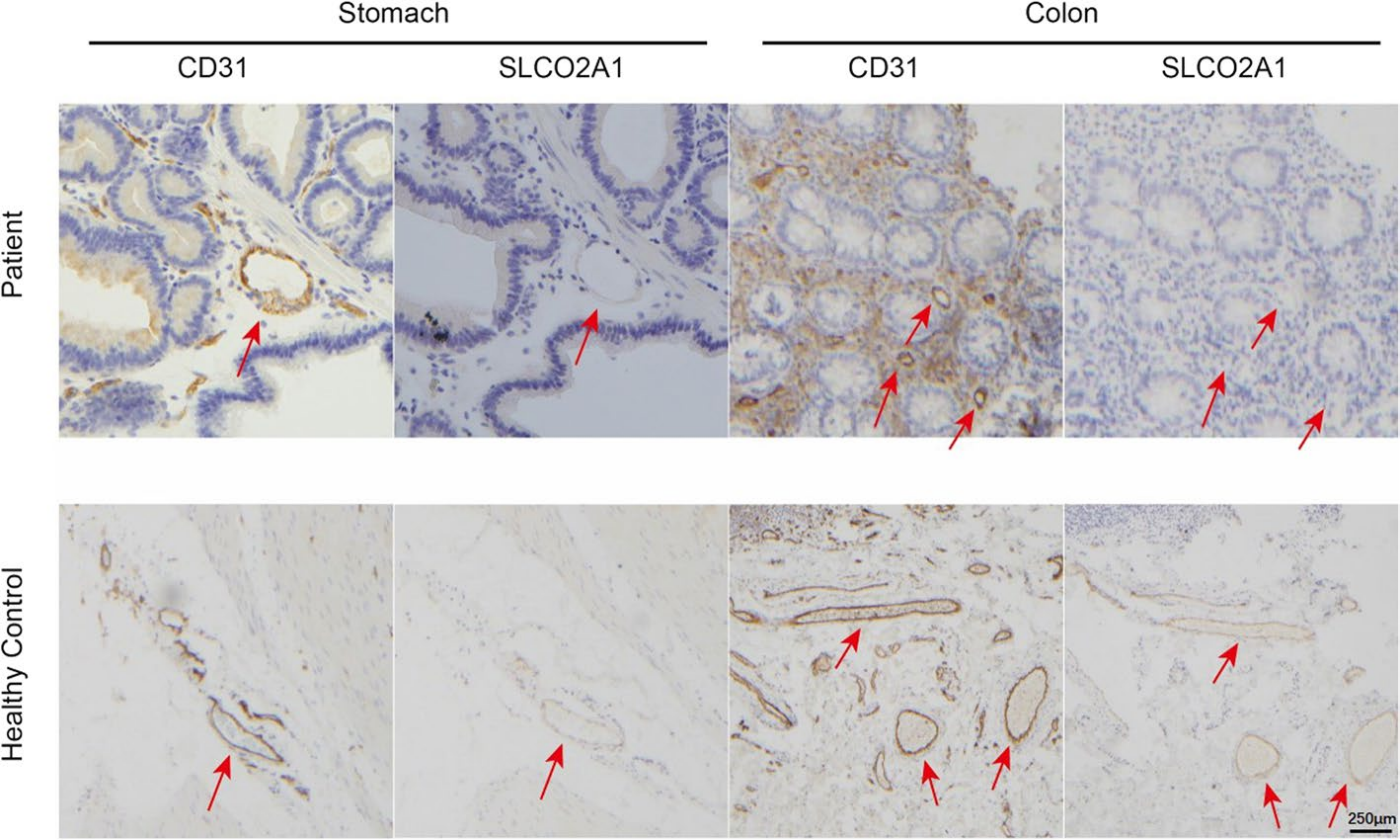
Immunohistochemistry of SLCO2A1 and CD31 in gastrointestinal mucosa of CEAS patient and healthy control (HC) (200× magnification). Serial sections of the patient and HC stomach and colon mucosa were stained for endothelial cell marker CD31 and SLCO2A1. Red arrows indicated mucosal vascular endothelium. In the HC, both CD31 and SLCO2A1 were expressed in endothelial cells. However, in the CEAS patient, SLCO2A1 expression was absent in endothelial cells positive for CD31 staining in both stomach and colon tissues

Quantitative PCR analysis of extracted RNA revealed a significant reduction in *SLCO2A1* expression in the gastric antrum and sigmoid colon tissues of the patient when compared to the HC (Fig. 7a, b). T-A cloning verification found no normal *SLCO2A1* transcripts in the patient’s gastric or colonic tissues. Sequencing results indicated a lack of signal for most transcripts, suggesting the presence of nonsense-mediated mRNA decay (NMD) (Fig. 8). In contrast, most transcripts from the HC’s gastric and colonic biopsies exhibited normal patterns. IHC staining using anti-CD31 as the primary antibody effectively labeled vascular endothelial cells in the gastrointestinal tissues. Moreover, there were some other transcripts, which explained the faint shorter bands in both the patient and HC lanes of the minigene RT-PCR products in Fig. 6. However, staining with anti-SLCO2A1 yielded negative results in the CD31-positive mucosal vascular endothelial cells of the patient, in contrast to the strong SLCO2A1 staining observed in the same cells of the HC (Fig. 9 & Supplementary Fig. 3).

## Discussion

In this study, we have reported a novel variant, c.929A>G, within the *SLCO2A1* gene. This variant was identified in a patient presenting with both CEAS and PHO. Notably, the c.929A>G variant is characterized by a single nucleotide substitution in exon 7, resulting in abnormal mRNA splicing and the deletion of 16 base pairs. This aberrant splicing is likely to activate nonsense-mediated mRNA decay (NMD). Intriguingly, despite the variant’s location within an exon, it does not manifest as a missense mutation, which typically leads to amino acid substitution as predicted by software. Instead, it exerts its effect on splicing, as demonstrated in our step-by step verification process. It is also important to note that prior reports have documented several variants located within exons of *SLCO2A1* in CEAS patients [7, 8, 10–12], which were classified solely based on software prediction as missense or nonsense mutations. Our findings underscore the necessity of concrete verification to avoid potential misinterpretation. In summary, our research has uncovered the novel exonic splicing variant, c.929A>G, shedding light on its potential role in the underlying pathogenic mechanisms of the disease.

The patient featured in our study initially presented with PHO and later developed gastrointestinal symptoms. His PHO diagnosis was confirmed based on typical clinical manifestations and genetic testing. However, unlike the classical reports of CEAS [10, 11, 13], he did not exhibit evident intestinal lesions. Nevertheless, the presence of significantly thickened gastric folds and duodenum indicated involvement of the upper GI tract, aligning with CEAS characteristics. Additionally, the mucosal lesions in the patients’ colon and rectum closely resembled those typically observed in ulcerative colitis. This resemblance was further confirmed by the positive response to mesalazine treatment. As a result, we considered the concurrent presence of UC as a comorbidity alongside CEAS and PHO in this patient.

Our investigation has delved into how this novel variant affects splicing. Next-generation sequencing identified an *SLCO2A1* variant, with Sanger sequencing confirming its homozygosity and maternal inheritance. Minigene analysis demonstrated abnormal in vitro transcription compared to the healthy control (Fig. 5a). qPCR analysis revealed reduced transcript levels and shortened transcript lengths in the patient, indicating that the variant diminishes both the quantity and length of the transcripts. Sequencing of the qPCR products allowed for the identification of the specific deleted region, suggesting potential changes in splicing sites (Fig. 6). In vivo analysis between the gastric and colonic tissues of the patient and HC showed more substantial differences in transcript levels, suggesting transcriptional disparities between in vitro and in vivo environments (Fig. 7). Furthermore, T-A cloning confirmed in vivo NMD, offering an explanation for the observed differences in mRNA levels (Fig. 8).

The precise mechanisms by which *SLCO2A1* variants lead to CEAS remain elusive. It is plausible that abnormal *SLCO2A1* expression disrupts the transmembrane transport and metabolism of PGE2, consequently causing an imbalance of PGE2 [1, 11]. However, the mechanisms responsible for the diverse clinical presentations and varying severities observed among patients with different variants remain poorly understood. Some patients exhibit both PHO and CEAS, while others manifest only one of these conditions. Additionally, CEAS patients present with varying involvement of the GI tract, as well as exhibit varying degrees of symptom severity [4, 7, 11]. Pathogenic variants in *SLCO2A1* can disrupt its function at multiple levels, encompassing transcription, translation, membrane localization, and PGE2 transport. These distinct mechanisms may contribute to the heterogeneous clinical manifestations and severities observed in patients. For instance, specific variants may lead to the production of truncated proteins, while others result in nearly complete proteins with single amino acid substitutions [4]. Investigating the correlation between the types of variants and their clinical manifestations warrants further study.

Our study has several limitations. Firstly, in both lanes of the qPCR gel (Fig. 7), two bands were observed in both the patient and the healthy control. The brighter bands were considered as target bands, and their identities were further confirmed by Sanger Sequencing. However, the dimmer bands were not recycled and sequenced individually. Although the specific sequences of the cDNA from the dimmer bands cannot be definitively identified, we conducted T-A cloning to explore the transcripts from the gastric and colonic tissues. This revealed several different transcripts beyond the target ones. The presence of these extra bands suggested the possibility of abnormal transcripts. Secondly, we were unable to identify the variants in the patient’s father due to his early death. Consequently, the source of the patient’s variant on paternal allele could not be definitively verified. However, both WES and Sanger Sequencing supported the homozygosity of the variant, providing partial support for a paternal source.

## Conclusion

In summary, we have identified a novel *SLCO2A1* mutation, c.929A>G, in a patient presenting with both PHO and CEAS. This genetic anomaly is likely responsible for the patient’s clinical manifestations and provides valuable insights for diagnosis and further exploration of potential pathogenic mechanisms associated with this mutation. Our findings contribute to the ongoing investigation into the pathogenic mechanisms underlying such mutations.

### Supplementary Information

Supplementary Material 1.

## Data Availability

The datasets used for analysis of current study are available on PubMed, EMBASE, Cochrane Library, Gene Bank of NCBI (https://www.ncbi.nlm.nih.gov/gene) and the China National Knowledge Infrastructure (www.cnki.net) database from January 1, 2000 to July 31st, 2023.
