# Supplementary material for: A novel variant in the SLCO2A1 gene in a Chinese patient with chronic gastroenteropathy and primary hypertrophic osteoarthropathy
Source: Orphanet J Rare Dis. 2024 Jun 11;19:229. doi: 10.1186/s13023-024-03221-x (PMC11165838; doi:10.1186/s13023-024-03221-x)
Supplement: Supplementary file 1 — Supplementary Material 1. [file 13023_2024_3221_MOESM1_ESM.pptx]

## Slide 1
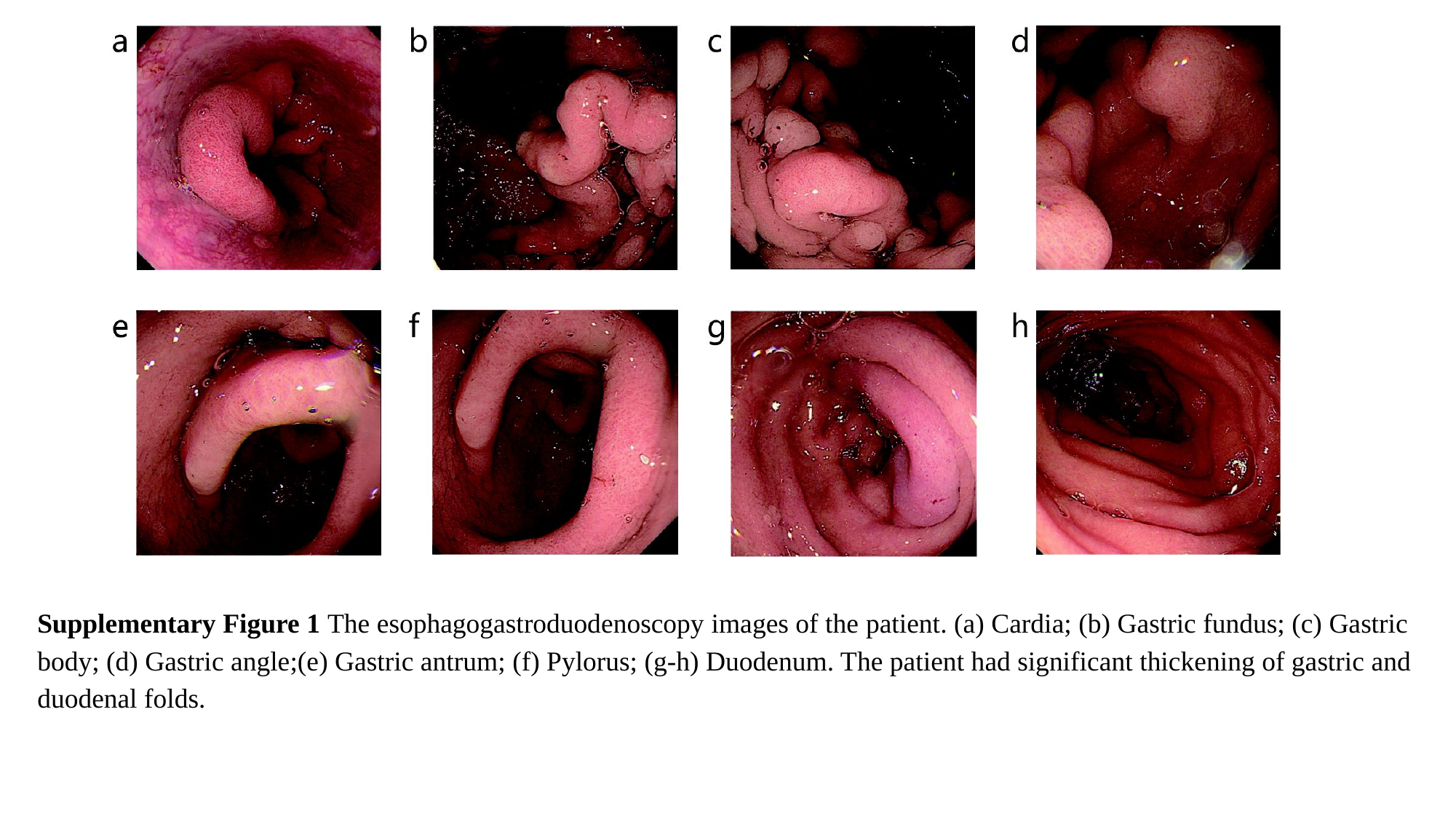

Supplementary Figure 1 The esophagogastroduodenoscopy images of the patient. (a) Cardia; (b) Gastric fundus; (c) Gastric body; (d) Gastric angle;(e) Gastric antrum; (f) Pylorus; (g-h) Duodenum. The patient had significant thickening of gastric and duodenal folds.

## Slide 2
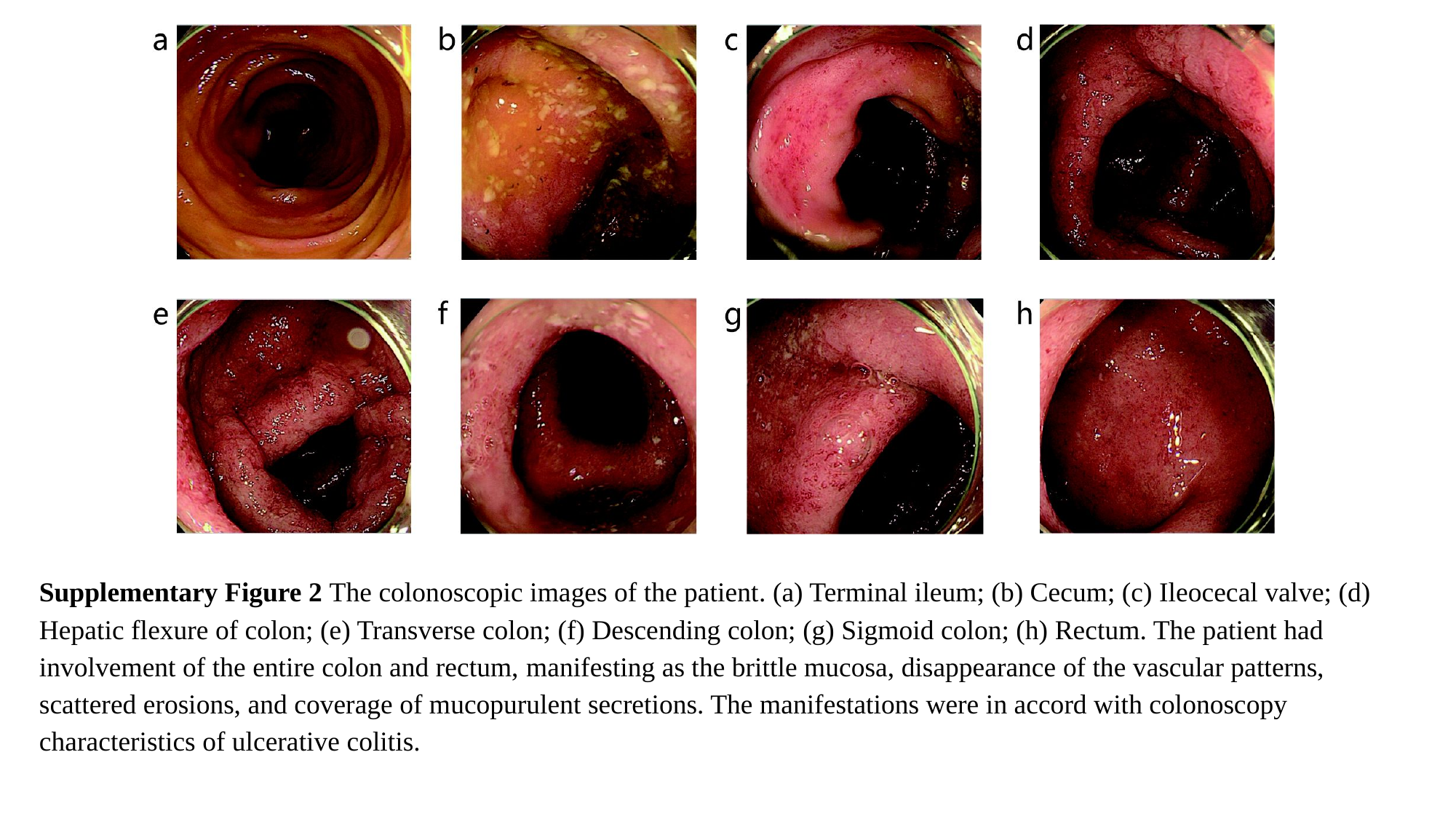

Supplementary Figure 2 The colonoscopic images of the patient. (a) Terminal ileum; (b) Cecum; (c) Ileocecal valve; (d) Hepatic flexure of colon; (e) Transverse colon; (f) Descending colon; (g) Sigmoid colon; (h) Rectum. The patient had involvement of the entire colon and rectum, manifesting as the brittle mucosa, disappearance of the vascular patterns, scattered erosions, and coverage of mucopurulent secretions. The manifestations were in accord with colonoscopy characteristics of ulcerative colitis.

## Slide 3
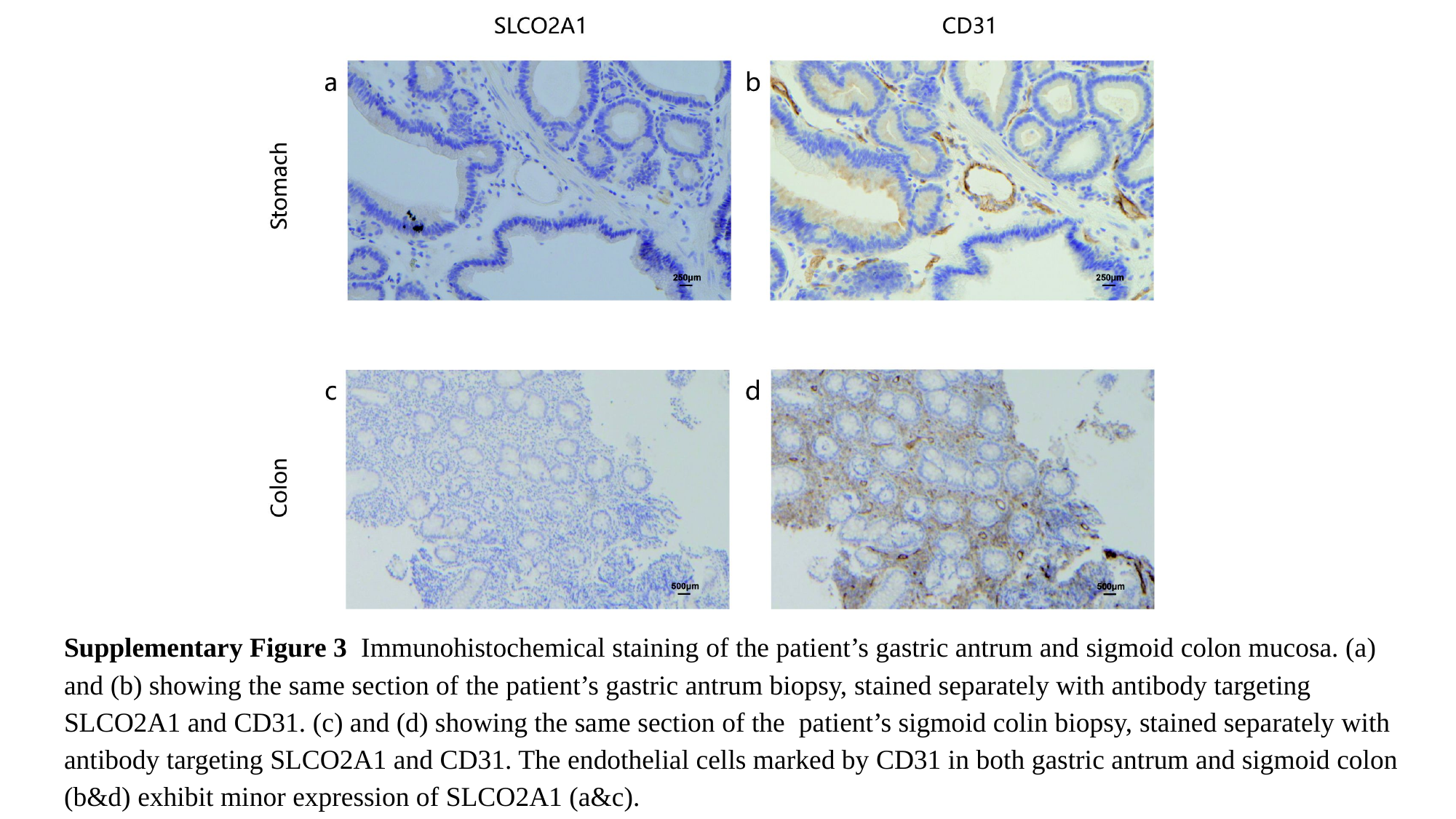

Supplementary Figure 3 Immunohistochemical staining of the patient’s gastric antrum and sigmoid colon mucosa. (a) and (b) showing the same section of the patient’s gastric antrum biopsy, stained separately with antibody targeting SLCO2A1 and CD31. (c) and (d) showing the same section of the patient’s sigmoid colin biopsy, stained separately with antibody targeting SLCO2A1 and CD31. The endothelial cells marked by CD31 in both gastric antrum and sigmoid colon (b&d) exhibit minor expression of SLCO2A1 (a&c).
